# Scientific Items

**Published:** 1882-11-20

**Authors:** 


### SCIENTIFIC ITEMS.

**The Ways of Plants.**—In a great many cases leaves are said to sleep; that is to say, at the approach of night they change their position, and sometimes fold themselves up, thus presenting a smaller surface for radiation, and being in consequence less exposed to cold. Mr. Darwin has proved experimentally that leaves which were prevented from moving suffered more from cold than those which were allowed to assume their natural position. He has observed with reference to one plant, *Maranta arundinncea*, the arrow-root, a West Indian species allied to *Canna*, that if the plant has had a severe shock it can not get to sleep for the next two or three nights.

The sleep of flowers is, also, probably a case of the same kind, though, as I have elsewhere attempted to show, it has now, I believe, special reference to the visits of insects; those flowers which are fertilized by bees, butterflies, and other day insects, sleep by night, if at all; while those which are dependent on moths rouse themselves toward evening, as already mentioned, and sleep by day. These motions, indeed, have but an indirect reference to our present subject. On the other hand, in the dandelion (*Leontodon*), the flower-stalk is upright while the flower is expanded, a period which lasts for three or four days; it then lowers itself and lies close to the ground for about twelve days, while the fruits are ripening, and then rises again when they are mature. In the *Cyclamen* the stalk curls itself up into a beautiful spiral after the flower has faded.—*Pop. Science Monthly.*

THE London Times publishes a synopsis of some papers on the "tremors of the earth" by the committee appointed to measure the lunar disturbance of gravity, and by Mr. G. Darwin, which contains some statements new to the public. It is considered proved by the men of science engaged that the crust of the earth bends under the weights imposed on it till, "when the barometer rises an inch over a land area like that of Australia, the increased load of air sinks the entire continent two or three inches below the normal level." The land actually sinks and rises under the pressure of the mass of water thrown upon it by the tides, the maximum rise and fall on the Atlantic seaboard reaching five inches. This effect is felt at the bottom of the deepest mine, and may reach for an unknown distance. It follows that the crust of the earth must be of exceeding tenacity, exceeding as a minimum that of granite; and its swayings may be the causes of phenomena hitherto quite unexplained, as, for example, the relation between storm and earthquake. So universal, frequent and unavoidable are these disturbances that the inquiry into the lunar disturbance of gravity has been given up. No depth can be found at which a recording instrument can be placed so as to escape their effect. The round earth pants, in fact, like a breathing being, under the changes always going on above her.—*Mech. News.*

CAUTIOUS—We are not sufficiently alive in this part of the globe to the importance of the maxim, "In time of peace prepare for war." Nobody has thought, for instance, of adding to the other hindrances to the completion of the Brooklyn Bridge a suggestion that whenever England invades this country she will take possession of Long Island, occupy Brooklyn and rush her troops over the bridge into New York. But it appears to be a settled fact that the project of a Channel Tunnel from England to France is to be abandoned for a reason very like the one we have described. General Sir Garnet Wolseley, who is just now in a position not to be safely contradicted, declares that it will not do. The Duke of Cambridge, commander-in-chief of the British army, is of the same opinion. A committee of which Sir Archibald Alison is the chairman has reported various plans for fortifying the tunnel, stifling a hostile force with irrespirable gas, drowning and crushing them, and blowing both them and the tunnel into the next or the nether world; but after all these grim precautions are taken, the committee still doubts whether the enemy might not get over. It follows that this generation will not witness the building of the tunnel; and it will fortunately pass away too soon to hear the derisive laughter of a future one.—*Mech. News.*

**Novel Uses of the Electric Light.**—A company is now negotiating with the government for a contract to light the City of Washington by placing around the dome of the Capitol a series of powerful electric lamps, aggregating several hundred thousand candles in brilliancy. It is proposed in this way to light the city to the distance of a mile in all directions better than it is usually done by street gas-lamps.

Pearl-fishing, it is now thought, can be conducted with great success by means of submerged electric lights in place of the old mode of employing divers. Incandescent lamps, of the Edison form will be let down to the ocean bed, making it as light as the surface in daylight, while operators with suitable grappling tongs, at the surface, will pick up the pearl oysters and deposit them in crates sunken for the purpose at the depth of a hundred feet or more.

Look out, now, for a supply of these lovely gems, larger than have yet been seen, since aged oysters can be taken from a depth far beyond the reach of the old-time diver.—*Literary Microcosm.*

MONS. CAMILLE FLAMMARION, the French astronomer, endeavors to account for the recent remarkable changes of Mars by supposing that inundations and other movements of great bodies of water are more extensive on that planet than on earth.—*Ex.*

THE transit of Venus, which occurs December 6, will be visible to the naked eye. The beginning and close of the transit can be seen in all the Atlantic States of North America, and throughout South America.—*Ex*

